# Wheat Pasta Enriched with Green Coffee Flour: Physicochemical, Antioxidant and Sensory Properties

**DOI:** 10.3390/molecules30244765

**Published:** 2025-12-13

**Authors:** Dariusz Dziki, Grażyna Cacak-Pietrzak, Julia Kopyto-Krzepicka, Agata Marzec, Sylwia Stępniewska, Anna Krajewska, Wioleta Dołomisiewicz, Renata Nowak, Sebastian Kanak

**Affiliations:** 1 Department of Thermal Technology, University of Life Sciences in Lublin, 31 Głęboka Street, 20-612 Lublin, Poland; 2 Department of Food Technology and Assessment, Institute of Food Sciences, Warsaw University of Life Sciences, 159C Nowoursynowska Street, 02-776 Warsaw, Poland; grazyna_cacak_pietrzak@sggw.edu.pl (G.C.-P.); sylwia_stepniewska@sggw.edu.pl (S.S.); 3 Faculty of Food Technology, Warsaw University of Life Sciences, 159C Nowoursynowska Street, 02-776 Warsaw, Poland; 4 Department of Food Engineering and Process Management, Institute of Food Sciences, Warsaw University of Life Sciences, 159C Nowoursynowska Street, 02-776 Warsaw, Poland; agata_marzec@sggw.edu.pl; 5 Department of Food Engineering and Machines, University of Life Sciences in Lublin, 28 Głęboka Street, 20-612 Lublin, Poland; anna.krajewska@up.edu.pl; 6 Department of Pharmaceutical Botany, Medical University of Lublin, 1 Chodzki Street, 20-835 Lublin, Poland; wioleta.dolomisiewicz@umlub.edu.pl (W.D.); renata.nowak@umlub.edu.pl (R.N.); sebastian.kanak@umlub.edu.pl (S.K.)

**Keywords:** fortification, chlorogenic acid, color, texture, phenolic profile, microstructure, antioxidant properties, quality, convective and microwave-vacuum drying

## Abstract

This study aimed to evaluate the impact of green coffee flour (GCF) addition (2–8%) and drying method (convective versus microwave-vacuum drying) on the physicochemical, textural, and bioactive properties of pasta. Both factors were found to significantly influence the assessed parameters. Green coffee had no observable effect on the microstructure of convectively dried pasta, whereas microwave-vacuum drying caused visible cracks and a heterogeneous starch-protein matrix even at a 2% supplementation level. Microwave-vacuum-dried pasta exhibited a shorter optimal cooking time and higher water absorption compared with convectively dried samples, while increasing the level of GCF prolonged cooking time and increased cooking losses. Texture analysis revealed that convectively dried pasta showed decreased elasticity and cohesiveness with increasing GCF content, whereas microwave -vacuum-dried pasta maintained a relatively uniform texture regardless of supplementation. The incorporation of GCF enhanced the antioxidant capacity of pasta, with the most pronounced effect at 2% addition, while higher levels showed reduced benefits. Similarly, fortification increased the content of phenolic acids, particularly chlorogenic acid and its isomers, with convectively dried samples exhibiting higher levels than microwave-vacuum-dried pasta. Consumer acceptance was highest for convectively dried pasta without GCF and for samples containing 2%, while pasta with higher GCF levels or microwave-vacuum-dried samples received lower scores.

## 1. Introduction

Green coffee, the unroasted seed of *Coffea* spp., has in recent years attracted considerable scientific attention as a valuable source of phenolic compounds, particularly chlorogenic acid [1,2,3]. In the context of glucose metabolism regulation, chlorogenic acid inhibits the activity of carbohydrate-degrading enzymes, thereby slowing glucose absorption in the small intestine and reducing postprandial glycemic response [4]. Clinical studies have also shown that it exhibits high bioavailability [5] and contributes to improvements in glycemic control and insulin resistance in overweight individuals [6].

Meta-analyses indicate that supplementation with green coffee bean extract (GCFE) significantly contributes to reductions in body weight and obesity-related indices (BMI, waist circumference) compared to placebo [4,7]. Moreover, GCFE may reduce visceral fat levels and help prevent muscle damage following physical exertion [8]. Intake of GCFE has also been shown to significantly decrease both systolic and diastolic blood pressure without affecting heart rate; this hypotensive effect was more pronounced in individuals with elevated baseline blood pressure, while no significant changes were observed among female participants [9].

Furthermore, consumption of green coffee bean extract has been associated with reductions in total cholesterol and triglyceride levels [10]. Studies also highlight the strong antioxidant [11] and anti-inflammatory [12] properties of green coffee extracts.

The enrichment of cereal-based products with plant-derived ingredients has become an important trend in the food industry, aimed at enhancing their nutritional and functional value. Plant-based additives, such as dried materials, extracts, or powders obtained from fruits, vegetables, and herbs, serve as sources of bioactive compounds, including polyphenols, vitamins, dietary fiber, and minerals [13]. Their incorporation not only increases the antioxidant potential of food products but also positively affects their color, taste, and aroma. Furthermore, the use of plant additives contributes to the development of functional foods that respond to the growing consumer demand for a healthy lifestyle and sustainable food production.

Green coffee beans themselves are not palatable, as they lack the characteristic aroma and flavor developed during roasting. Therefore, in food fortification, green coffee bean flour obtained from ground unroasted beans can be used as a functional ingredient to enrich products with bioactive compounds, particularly chlorogenic acid, without imparting undesirable flavor. In most studies, scientific attention has been focused on green coffee extracts and their potential application in food fortification [11,14,15,16]. The incorporation of green coffee bean flour appears to be a more promising and sustainable trend than the use of pure green coffee extracts, as it allows the utilization of the whole raw material, preserves dietary fiber and other valuable components, and avoids the need for solvent-based extraction processes. Moreover, flour-based enrichment may offer better technological compatibility with cereal matrices, facilitating the development of clean-label and fiber-rich functional products. Green coffee bean flour has been used to enrich wheat bread [17,18,19] and gluten-free cakes [20]. In all cases, an increase in the total phenolic content of the final product was observed. Moreover, the addition of green coffee bean flour to bread significantly altered its physicochemical properties [21]. It was also demonstrated that residues remaining after chlorogenic acid extraction from green coffee could serve as valuable ingredients in shortbread cookies, improving not only their potential health-promoting value but also sensory attributes such as color and aroma [22]. To date, however, no studies have addressed the potential incorporation of green coffee into pasta formulations. Pasta ranks among the most widely consumed cereal products worldwide and constitutes a major component of the daily diet in many regions. Its popularity is attributed to its ease of preparation, long shelf life, and culinary versatility. Traditionally, pasta is produced from durum wheat semolina (*Triticum durum*) or wheat flour, ensuring desirable structural, sensory, and color properties. Nevertheless, despite being a valuable source of energy, traditional pasta contains relatively low amounts of bioactive compounds, dietary fiber, and several essential vitamins and minerals. Increasing consumer awareness of the relationship between diet and health has stimulated growing interest in functional foods, encouraging researchers and manufacturers to develop innovative strategies to improve the nutritional quality of traditional products, including pasta. The incorporation of plant-based materials, such as flours obtained from fruits and vegetables [23] and their by-products [24,25], algae [26], and other plant materials [27], into pasta has become a promising direction in modern food technology, combining nutritional innovation with sustainability and technological advancement.

Drying of pasta is a critical stage in its production, significantly influencing the quality of the final product. This process is essential not only for extending the shelf life of pasta but also for ensuring its proper structure, texture, and resistance to overcooking. Properly conducted drying allows pasta to maintain its shape, elasticity, and sensory attributes, including taste and appearance. Furthermore, careful control of the drying process minimizes the risk of microbial growth, which is crucial for food safety. For these reasons, pasta drying plays a vital role in the food industry and is a subject of extensive technological research [28,29,30]. Despite the critical role of drying in pasta production, the existing literature contains relatively few reports on alternative drying methods. Most studies and industrial practices focus primarily on high-temperature drying, while other techniques, such as low-temperature drying or vacuum drying, have received limited attention. This gap highlights the need for further research to evaluate the potential of different drying approaches in terms of pasta quality, nutritional properties, and processing efficiency.

The aim of this study was to investigate the potential of enriching wheat pasta with green coffee flour (GCF) to enhance its functional value by evaluating its effects on bioactive compound content, as well as physicochemical and sensory properties. Additionally, we studied the effects of different drying methods on these pasta characteristics.

## 2. Results and Discussion

### 2.1. Basic Chemical Composition of Raw Materials

Semolina (SE), used as the primary raw material for pasta production, exhibited significantly lower content of total protein, mineral (ash), fat, and dietary fiber compared to GCF, and higher digestible carbohydrate content (Table 1). GCF contained significantly more total protein than SE (17.59% versus 12.01%) on a dry matter basis (d.m.), although these proteins are predominantly storage proteins, such as globulins and albumins, and do not contain gluten proteins (gliadins and glutenins) [31]. In contrast, gluten proteins account for approximately 80–85% of the total protein in SE, conferring dough elasticity and viscosity and exerting a decisive influence on pasta quality [32]. Notably, GCF is characterized by a high dietary fiber content, amounting to over 22% on a dry matter basis. The main fraction of non-starch polysaccharides in raw coffee beans consists of galactomannans, followed by arabinogalactans, with smaller amounts of cellulose and hemicelluloses [33,34]. Dietary fiber plays a crucial role in regulating digestive processes, stimulating intestinal peristalsis, potentially contributing to reductions in blood glucose and cholesterol levels, supporting the development of beneficial gut microbiota, and prolonging postprandial satiety [35]. Quantitatively, digestible carbohydrates, mainly starch [36], dominate in semolina, whereas green coffee beans do not contain starch; their digestible carbohydrate fraction comprises primarily small amounts of sucrose [37].

### 2.2. Microstructure of Pasta

**Figure 1:**
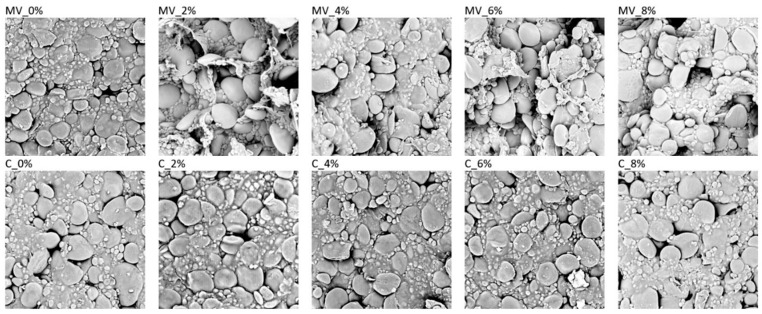
Scanning electron micrographs (2000× magnification) of pasta convectively dried (C) and microwave-vacuum-dried (MV), with the addition of different levels of green coffee flour (0, 2, 4, 6, and 8%; g/100 g sample).

The microstructure of pasta samples is presented in Figure 1. Starch granules of various sizes and predominantly round or oval shapes were observed. Small starch granules filled the protein matrix and surrounded the larger ones. Pores were present in all samples; however, the microstructure of the convectively dried pasta appeared more compact and less porous compared to the microwave-vacuum-dried pasta enriched with green coffee. The addition of GCF did not affect the microstructure of the convectively dried pasta, whereas microwave-vacuum drying, even with the lowest tested addition level of 2%, led to noticeable changes in the pasta microstructure. Distinct cracks were visible. As a result of microwave-vacuum drying, gluten combined with GCF formed a structure in which fine starch granules surrounded clusters of large starch granules (Figure 1), a phenomenon not observed in conventionally dried pasta. Similar differences in the microstructure of pasta dried by convective and microwave-vacuum methods were also reported in earlier studies [38]. As demonstrated, these differences influenced the rate of water absorption, the loss of dry matter during cooking, and the texture of the cooked pasta. This indicates that optimization of formulation or processing parameters may be necessary to preserve the desirable GCF-pasta texture when microwave-vacuum drying is used for pasta dehydration.

### 2.3. Culinary Properties of Pasta

The experimentally determined optimal cooking time of pasta, defined as the time required for the disappearance of the so-called white core in the pasta cross-section, was significantly affected by both the level of GCF addition and the drying method (Table 2). The optimal cooking time of pasta dried by microwave-vacuum drying ranged from 3.5 min (control sample) to 5.5 min (samples with 6 and 8% GCF) and was approximately twice as short as that of convectively dried pasta (7.0–11.5 min). The faster hydration of microwave-vacuum-dried pasta compared to convectively dried samples can be attributed to its more porous microstructure, resulting from the rapid evaporation of water vapor under reduced pressure during drying. The looser starch–protein matrix facilitates faster water penetration into the pasta and increases the contact surface area with water, thereby accelerating starch hydration and gelatinization [39,40].

The incorporation of GCF into pasta increased its cooking time, with statistically significant changes already observed at a 2% addition level. As the green coffee content increased, a linear rise in cooking time was noted. A similar trend was reported by Teterycz et al. [41] after enriching pasta with hemp seed flour. This effect may be associated with the increased fiber content in the pasta. Dietary fiber, due to its high water absorption capacity and competition with starch for water, hinders starch granule swelling and gelatinization, thus prolonging the cooking time (Table 2).

The weight increase index, representing the pasta’s ability to take up water during cooking, ranged from 2.5 (convectively dried pasta without GCF) to 3.3 (microwave-vacuum-dried pasta with 8% GCF) (Table 2). Both the GCF addition level and the drying method had a statistically significant effect on this parameter. Convectively dried pasta exhibited a lower mass increase compared to microwave-vacuum-dried samples, consistent with previous findings [38]. The higher values obtained for microwave-vacuum dried pasta can be explained by its more porous microstructure, which enhances water absorption during cooking [40]. The incorporation of green coffee into pasta also increased water absorption. In convectively dried samples, statistically significant differences relative to the control were observed at 6% and 8% additions, while in microwave-vacuum-dried pasta, significance occurred at 8% GCF addition. This effect may result from the weakening of the protein matrix caused by the addition of a high-fiber and high-protein ingredient lacking gluten-forming proteins. A similar pattern was reported by Teterycz et al. [41] when enriching pasta with hemp seed flour.

The cooking loss of dry matter ranged from 5.10% (convectively dried pasta without GCF) to 10.40% (microwave-vacuum-dried pasta with 8% GCF) (Table 2). This parameter was significantly affected by both the level of GCF addition and the drying method. Convectively dried pasta showed lower cooking losses compared to microwave-vacuum-dried samples, which could be attributed to its more compact microstructure that limits the leaching of nutrients during cooking. The addition of GCF increased cooking losses. A similar tendency was reported by Teterycz et al. [41] after enriching pasta with hemp seed flour. This effect may be explained by the weakening of the protein matrix caused by the gluten-free additive and by the higher content of water-soluble components, which are more easily leached out during cooking.

From a practical perspective, these findings have important implications for industrial pasta production and consumer convenience. Microwave-vacuum drying can significantly reduce cooking time, which may enhance energy efficiency and throughput in industrial settings, while also offering convenience to consumers seeking quicker meal preparation. However, the addition of GCF, especially at higher levels, prolongs cooking time and increases cooking losses, indicating the need to balance functional enrichment with consumer expectations for fast-cooking pasta. Optimization of both formulation and processing conditions may therefore be necessary to achieve a product that combines nutritional enhancement, desirable texture, and practical usability.

### 2.4. Color of Raw Materials and Pasta

SE and GCF differed significantly in color parameters (Table 3). The L* values, describing color lightness, were 84.57 for SE and 71.65 for GCF. GCF exhibited significantly higher redness than SE (2.16 and 1.66, respectively), whereas SE showed significantly higher yellowness (22.31 and 16.42, respectively). The yellow color of semolina results from its high carotenoid content, particularly lutein and β-carotene [42]. The greenish-yellow color of green coffee beans results primarily from the presence of chlorophylls (a* and b*) and carotenoids such as lutein and β-carotene [43].

The color of pasta plays a key role in shaping consumer acceptance. Light yellow pasta is particularly preferred [44]. The factors determining pasta color include the color of the raw materials used in its production and the processing technology, especially the drying conditions [45,46]. The color parameters of the examined pasta (L*, a*, b*) were significantly influenced both by the addition of GCF and by the drying method applied (Table 4). Convectively dried pasta was slightly darker than microwave-vacuum-dried pasta, with a higher proportion of red and yellow hues. A similar effect of the drying method on pasta color was observed in a previous study [38], in which pasta was enriched with *Rosa rugosa* fruit pomace. These differences may result from the higher temperature, significantly longer duration, and greater oxygen exposure during conventional drying, likely leading to non-enzymatic browning and, consequently, a darker product color. The incorporation of GCF into the pasta caused a darkening of its color, noticeable even at the lowest 2% addition level. As the green coffee content increased, lightness decreased, redness increased, and yellowness decreased. These changes were due to the differences in color between semolina and green coffee beans (Table 3). From a practical standpoint, such color modifications may affect consumer acceptance and marketability, as darker pasta or pasta with reduced yellowness may be perceived as less typical or less appealing. Therefore, producers incorporating GCF should consider strategies to balance the desired functional benefits with visual attributes that align with consumer expectations.

### 2.5. Texture of Pasta

Texture is one of the fundamental criteria for assessing pasta quality, directly influencing consumer acceptance. Pasta texture depends on numerous interrelated factors, including the properties of the raw materials (e.g., water absorption capacity, strength and continuity of the gluten network, degree of starch gelatinization, presence of non-gluten components) as well as processing conditions, particularly drying and cooking parameters [47,48]. The elasticity and cohesiveness of the examined pasta were significantly affected by both the drying method and the level of GCF addition, whereas springiness was influenced solely by the drying method (Table 5). Elasticity, which measures the ability of pasta to return to its original shape after the removal of a deforming force, ranged from 0.15 (convectively dried pasta with 8% GCF) to 0.35 (convectively dried pasta without GCF). For convectively dried pasta, elasticity gradually decreased with increasing GCF content. This trend was not observed in microwave-vacuum-dried pasta, which exhibited similar elasticity values regardless of GCF addition, ranging from 0.21 to 0.23. Springiness ranged from 0.39 (convectively dried pasta with 8% GCF) to 0.84 (convectively dried pasta without GCF). Similarly to elasticity, microwave-vacuum-dried pasta showed no significant variation in springiness, with values between 0.52 and 0.62. Cohesiveness, describing the ability of pasta to maintain its shape during cooking, ranged from 0.36 (convectively dried pasta with 8% GCF) to 0.71 (convectively dried pasta without GCF). For microwave-vacuum-dried pasta, cohesiveness values did not differ significantly, with values ranging from 0.61 to 0.66. The differences in pasta texture between convectively and microwave-vacuum-dried samples, as well as the effect of GCF addition, can be attributed to the interplay of drying conditions and raw material composition. Conventional drying, with higher temperatures, longer duration, and greater oxygen exposure, likely disrupted the gluten network and partially denatured proteins, reducing elasticity and cohesiveness. In contrast, microwave-vacuum drying preserved gluten integrity, resulting in more uniform texture regardless of GCF level. The addition of GCF, rich in fiber and polyphenols, interfered with gluten cohesion and water absorption, particularly in convectively dried pasta, while its impact was minimal under gentle microwave-vacuum drying conditions. The drying technique affected the microstructure of the pasta (Figure 1), which likely determined its textural properties. In the microwave-vacuum-dried pasta, the microstructure was less compact compared with conventionally dried pasta. In our other studies, we demonstrated that conventionally dried pasta had significantly lower porosity but a greater number of pores with large surface areas compared with microwave-vacuum-dried pasta [38]. Lin et al. [49] reported that gluten in samples dried using microwave and dielectric methods formed a dense network with a distinct cross-linked structure, covering nearly all starch granules. Moreover, Altan and Maskan [50] showed that the degree of starch gelatinization was higher in pasta dried with hot air at 62 °C than in pasta dried using microwaves at 70 and 210 W. This may explain the shorter hydration time of the pasta and may influence its water-holding capacity, and consequently, its texture.

### 2.6. Total Polyphenols and Antioxidant Capacity

GCF contained more than sixty times higher levels of phenolic compounds than semolina. Moreover, its antioxidant capacity was considerably greater. In addition, the effective concentration of GCF required to reduce 50% of ABTS and DPPH free radicals (EC_50_ index) was approximately 15 and 30 times lower, respectively, compared to SE, indicating a markedly higher antioxidant activity of GCF (Table 6). Consequently, both the phenolic compound content and antioxidant activity of the pasta increased. The incorporation of only 2% GCF resulted in more than a twofold increase in total phenolic content, while pasta containing 8% GCF exhibited approximately a sixfold higher polyphenol level (Table 7). The total phenolic content increased proportionally with the GCF addition (r = 0.998, *p* < 0.001).

Similarly, the introduction of GCF into pasta enhanced its ability to neutralize free radicals, as reflected by the lower EC_50_ values obtained in both the ABTS and DPPH assays (Table 7). The greatest changes in this parameter were observed at the 2% supplementation level. This effect may be attributed to the optimal concentration of bioactive compounds introduced with GCF, which was sufficient to enhance the antioxidant potential of the pasta without adversely affecting its matrix structure or the extractability of phenolic compounds. Higher supplementation levels might have led to interactions between phenolic compounds and macromolecules such as proteins or starch, thereby limiting their antioxidant availability. This effect may also reflect a typical nonlinear dose–response relationship with diminishing marginal gains. Furthermore, pronounced physico-structural changes observed in the pasta matrix could influence both the extractability and accessibility of phenolic compounds, contributing to the reduced measured antioxidant activity at higher supplementation levels. Similar trends were reported by Zain et al. [51], who fortified wheat bread with green coffee flour. In their study, polyphenol content increased proportionally with the level of green coffee addition (3–7%), while the most pronounced improvement in antioxidant activity was observed at 3%, with higher levels exhibiting a diminished effect. These findings confirm that moderate enrichment levels can maximize the antioxidant potential of cereal-based foods without compromising product quality. Despite statistically significant differences between some mean values, hot-air drying and vacuum drying produced pasta with similar phenolic content and antioxidant activity. The main factor determining these properties was the addition of GCF. Although convective drying and microwave-vacuum drying produced statistically significant differences between some mean values, the phenolic content and antioxidant activity of the pasta were similar, indicating that the main factor determining these properties was the addition of GCF. At the same time, it is important to consider the bioavailability of these phenolic compounds after cooking, as thermal processing may modify their structure or release bound fractions, thereby affecting their absorption and actual physiological efficacy. Consequently, the potential health benefits of GCF-enriched pasta will depend not only on its phenolic content and in vitro antioxidant potential, but also on the extent to which these compounds remain bioavailable following cooking. Future studies assessing in vitro digestion or bioaccessibility would help clarify the nutritional relevance of the observed increases in antioxidant potential.

### 2.7. Results of Phenolic Acids Identification

In semolina, the following phenolic acids were identified: chlorogenic, crypto-chlorogenic, neochlorogenic, and *p*-coumaric acids. In contrast, flour derived from green coffee beans also contained caffeic, protocatechuic, salicylic, and gallic acids (Table 8). Among all detected compounds, chlorogenic acid was present in the highest concentration in both semolina and GCF. However, its content in GCF was approximately four times higher. Moreover, the GCF exhibited substantially greater amounts of other phenolic acids, particularly neochlorogenic and crypto-chlorogenic acids, whose concentrations were approximately 63 and 32 times higher, respectively, compared to those found in semolina. Chlorogenic acid and its structural isomers, neochlorogenic and cryptochlorogenic acids, are among the most abundant phenolic compounds found in plant-derived materials such as coffee beans, apples, and cereals. These compounds belong to the group of *caffeoylquinic acids*, which are esters formed between caffeic acid and quinic acid. These acids are recognized for their strong antioxidant properties, as they effectively scavenge free radicals and inhibit lipid oxidation. In addition, they exhibit anti-inflammatory, antidiabetic, and cardioprotective activities, making them valuable bioactive constituents in functional foods [52]. GCF, in particular, is known to contain significantly higher levels of chlorogenic and related isomeric acids compared to semolina, which may enhance its overall antioxidant potential [53].

Fortification of pasta with GCF resulted in an increased content of phenolic acids, particularly chlorogenic acid and its structural isomers. Enriched pasta samples also exhibited the presence of caffeic, protocatechuic, and gallic acids, along with a rise in *p*-coumaric acid content. In most cases, the levels of these acids increased proportionally with the addition of green coffee (Table 9). Such tendencies were observed for both convectively and microwave-vacuum-dried pasta. The drying method also significantly influenced the phenolic profile, with convectively dried pasta generally showing higher phenolic acid concentrations. This effect is likely associated with the greater release of bound phenolics under higher temperature and prolonged exposure conditions, which can disrupt cell wall structures, liberate phenolic compounds bound to polysaccharides and proteins, and enhance their extractability during analysis—a phenomenon also reported for various food matrices, where elevated drying temperatures or hot-air drying increase the concentration of phenolic acids [49,50].

### 2.8. Sensory Properties of Pasta

**Figure 2:**
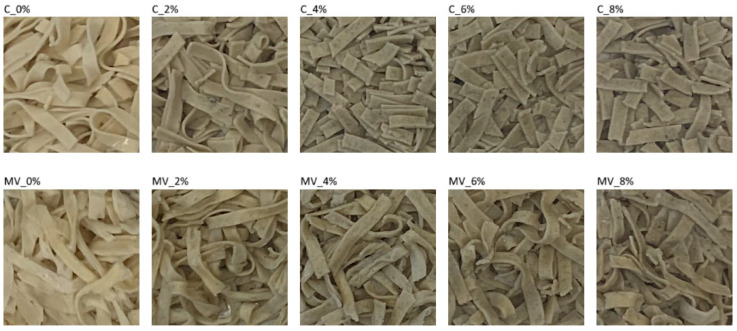
Cooked pasta convectively dried (C) and microwave-vacuum dried (MV) and with the addition of different levels of green coffee flour: 0, 2, 4, 6, and 8% (g/100 g SE).

Sensory evaluation constitutes a fundamental component of food quality assessment, as it determines how consumers perceive a product with respect to attributes such as taste, aroma, color, texture, and overall acceptability. Even when a product meets technological and nutritional standards, it is the sensory experience that ultimately shapes its reception and market potential. Modifications in formulation, including the incorporation of novel functional ingredients, as well as changes in processing methods, may substantially influence product perception; therefore, their evaluation is essential for determining the acceptability of the final product. In the present study, a sensory evaluation was performed on pasta enriched with green coffee flour (GCF) and dried using different methods. The results of this evaluation are presented in Table 10, while photographs of the cooked samples are shown in Figure 2. All evaluated attributes—taste, smell, color, texture, and overall acceptability—were significantly affected by the drying method. The addition of GCF influenced most of these characteristics, except for smell. Overall, pasta dried using the convective method exhibited higher sensory acceptability than that obtained by the microwave-vacuum method. The incorporation of GCF altered the taste of the pasta; as the level of this additive increased, a more pronounced sour–bitter, slightly astringent, and raw flavor reminiscent of green tea was perceived. The flavor profile of green coffee beans differs from that of roasted coffee, as it is shaped by natural plant constituents, primarily chlorogenic acids, alkaloids (caffeine and trigonelline), and organic acids (citric and malic acids) [54]. The addition of GCF also modified the smell of the pasta, imparting a plant-like note, sometimes described as “grassy” (Table 10). Green coffee beans contain more than 200 volatile compounds, although most occur at very low concentrations. The major compounds responsible for the smell of green coffee are aliphatic aldehydes and alcohols [55].

Several technological approaches may help mitigate the undesirable smell and taste of pasta enriched with GCF. Combined drying strategies can reduce the thermal release of volatile compounds, thereby diminishing their impact on the final sensory profile. Encapsulation of GCF and the use of brief steam treatment prior to incorporation may also decrease the intensity of grassy notes by limiting the release of volatiles or promoting partial degradation of odor-active molecules. Additionally, pairing GCF with complementary flavoring ingredients (e.g., mild cereal or herb extracts) may help mask the green smell and sour–bitter notes without altering the functional properties of the pasta. Therefore, methods aimed at reducing or masking the grassy smell and sour–bitter taste of GCF-enriched pasta should be considered an important direction for future research.

Regarding color, even a 2% addition of GCF caused a noticeable change from light cream to greenish-gray, which was perceived as less appealing by consumers. The dominant pigments in unroasted coffee beans are chlorophylls, which impart a greenish hue to both the beans and the products containing them [56]. The texture of the pasta was also affected, with firmness decreasing and stickiness increasing. A similar trend following the inclusion of various gluten-free plant additives has been observed in previous studies by the authors [38].

In summary, the highest overall acceptability was observed for convectively dried pasta without GCF and for samples with a 2% incorporation level. Convectively dried pasta containing 8% GCF, as well as microwave-vacuum-dried pasta with 6% and 8% of the additive, did not achieve consumer acceptance.

### 2.9. Principal Components Analysis

**Figure 3:**
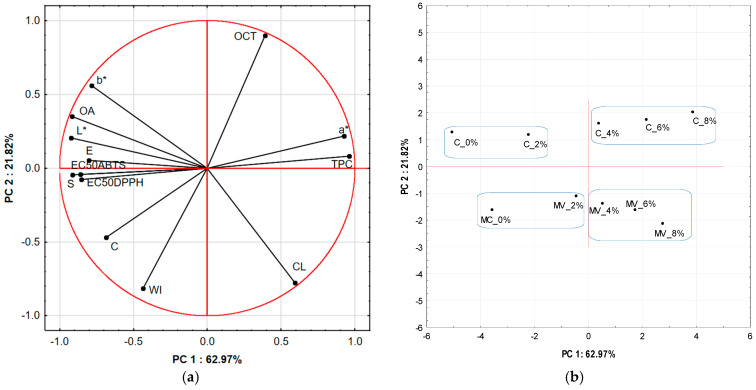
Principal component analysis (PCA) of physicochemical and sensory parameters of pasta samples: (**a**) PCA loading plot of two principal components PC1 and PC2, (**b**) score plot presenting analyzed pasta samples in terms of PC1 vs. PC2. Points on graph (**b**) enclosed within blue loops represent samples with similar values of the examined indicators. Explanations: sample codes C and MV refer to convectively and microwave-vacuum dried pasta, respectively; 0%, 2%, 4%, 6%, and 8% indicate the addition of different levels of green coffee flour: 0, 2, 4, 6, and 8% (g/100 g SE). Variable codes: OCT—optimal cooking time, WI—weight increase index, CL—cooking loss, L*, a*, b*—color parameters, E—elasticity, S—springiness, C—cohesiveness, OA—overall acceptability, TPC—total phenolic content, EC—antioxidant activity (EC_50_ for DPPH and ABTS).

Principal component analysis (PCA) was performed to determine the relationships between the physicochemical and sensory parameters of pasta dried by two methods: convective (C) and microwave-vacuum (MV), with the addition of 0–8% GCF. The first two principal components (PC1 and PC2) together explained 84.79% of the total data variability, with PC1 accounting for 62.97% and PC2 for 21.82% (Figure 3). The first component (PC1) primarily differentiated the samples in terms of total phenolic content (TPC), antioxidant activity (EC), color parameters (L*, a*, b*), texture parameters such as springiness (S) and elasticity (E), as well as overall acceptability (OA). High positive PC1 values were associated with higher phenolic content (TPC) and more intense coloration towards red (higher a* values). In contrast, negative PC1 values corresponded to higher lightness (L*), higher S and E parameters, and greater OA. The second component (PC2) primarily differentiated the samples based on cooking related parameters, including optimal cooking time (OCT), weight increase index (WI), and cooking loss (CL). In the loading plot (Figure 3a), the EC vectors (antioxidant activity, expressed as EC_50_ for DPPH and ABTS radicals) were oriented opposite to TPC, indicating that higher phenolic content resulted in lower EC_50_ values and thus higher antioxidant activity. The score plot (Figure 3b) revealed distinct clusters corresponding to the drying method. The convective (C) samples occupied the area with positive PC2 values, while the microwave-vacuum dried (MV) pasta samples were located on the negative side of PC2. Within both drying groups, a systematic shift in samples along the PC1 axis was observed with increasing coffee addition (0–8%), reflecting the enhancement of phenolic content, antioxidant activity, and more intense red coloration of the pasta. These findings indicate that both the drying method and the coffee level significantly affected the quality profile of the pasta, with the fortification effect being more pronounced in the air-dried samples. From a practical perspective, the PCA results help identify key parameters that can be used to optimize pasta formulation and drying conditions, thereby achieving improved antioxidant properties, a desirable color, and favorable textural and sensory characteristics.

## 3. Materials and Methods

### 3.1. Materials

Commercial SE from *Triticum durum* wheat (Polskie Młyny Sp. z o.o., Nakło, Poland) and Robusta GCF marketed under the trade name India Cherry AA/AB (NKG India Coffee Pvt. Ltd., Bengaluru, India) were used for pasta preparation. The coffee beans were ground using a Grindomix GM 200 laboratory knife mill (Retsch, Düsseldorf, Germany) to a particle size below 0.5 mm, placed in a vacuum-sealed foil bag, and stored in a dark location until analysis.

The following chemical reagents were used in the study: Folin–Ciocalteu reagent, ABTS (2,2′-azino-bis(3-ethylbenzothiazoline-6-sulfonic acid)), DPPH (2,2-diphenyl-1-picrylhydrazyl), methanol, sodium carbonate, gallic acid standard, and standards of phenolic acids. All chemicals were purchased from Sigma-Aldrich (Poznań, Poland). Enzymes (α-amylase, protease, and amyloglucosidase) were supplied by Megazyme International Ireland Ltd. (Wicklow, Ireland).

### 3.2. Basic Composition of SE and GCF

The AACC [57] methods were used to analyze the chemical composition of SE and GCF. Moisture content was determined using the air-oven method. Samples of the raw material (2 g) were placed in a laboratory dryer SLW 15 (POL-EKO, Wodzisław Śląski, Poland) and dried at 130 °C for 60 min following AACC Method 44-15A. Total protein content was measured using a Kjeltec™ 8400 apparatus (Foss Analytical AB, Höganäs, Sweden). Distillation was carried out with the automatic Kjeltec Auto kit (Tecator, Höganäs, Sweden), and nitrogen content was converted to protein using the factor N × 5.7. Ash content was determined according to AACC Method 08-01. Samples (5 g) were placed in ash dishes and incinerated in a muffle furnace at 550 °C until light-gray ash or constant weight was obtained (approximately 7 h). After cooling, the samples were weighed and ash content was calculated. Total fat content was determined by continuous ether extraction using a Soxtec™ 8000 apparatus (Foss Analytical AB, Höganäs, Sweden) with hexane as the extraction solvent. The procedure, based on the principle of Soxhlet extraction, was performed in accordance with AACC Method 30-25.01. Samples were extracted until complete fat removal; the solvent was then evaporated, and the remaining fat residue was weighed. Total dietary fiber was determined using the enzymatic-gravimetric method (AACC 32-05). Samples (1 g) were sequentially digested with heat-stable α-amylase, protease, and amyloglucosidase. Digestible carbohydrates were calculated by difference [58].

### 3.3. Pasta Preparation

Pasta samples were prepared using semolina (SE) and water, as well as mixtures of SE with ground green coffee flour (GCF) and water. SE was partially replaced with GCF at levels of 2, 4, 6, and 8%. A constant amount of water was added to both SE and SE–GCF mixtures to achieve a dough moisture content of 38%. The dough was mixed using a T-5KPM5EER mixer (KitchenAid, Benton Harbor, MI, USA) for 5 min. It was then rolled into sheets 1.5 mm thick and cut into tagliatelle strips 6 mm wide using a pasta roller attachment 5KSMPSA (KitchenAid, Benton Harbor, MI, USA). The formed pasta was dried using two methods: convective drying and microwave-vacuum drying.

In the convective drying method, weighed pasta samples (100 ± 0.01 g) were placed on perforated trays and dried in a convective dryer SUP-65 (WAMED, Warsaw, Poland) at 60 °C for 7–8 h. The target moisture content after drying was 12.0% (±0.2%) [59]. In the microwave-vacuum drying method, weighed pasta samples (100 ± 0.01 g) were placed in the chamber of a laboratory microwave-vacuum dryer (Promis-Tech, Wrocław, Poland). Drying was carried out at 50 °C for 300 s under a pressure of 55 hPa and a microwave power of 450 W, followed by a stabilization phase lasting 200 s. The drying parameters were experimentally determined to achieve a final moisture content of 12.0% (±0.2%) [39]. After cooling, the dried pasta samples were packed in paper bags and placed in a tightly sealed plastic container. Until analysis, the samples were stored in the dark at 20 ± 2 °C.

### 3.4. Determination of Pasta Microstructure

Pasta strands approximately 5 mm in length were mounted on a sample holder and sputter-coated with gold (200 Å) using a Leica sputter coater (Leica Mikrosysteme, Vienna, Austria). The coating thickness was 5 nm. Microstructural observations were carried out using a Phenom ProX scanning electron microscope (Phenom-World BV, FEI Company, Eindhoven, The Netherlands) at an accelerating voltage of 10 kV and a magnification of 2000×. All measurements were performed in ten replicates.

### 3.5. Determination of Culinary Properties of Pasta

The optimal cooking time was determined experimentally as the minimum cooking duration required for the disappearance of the white core in the cross-section of the pasta. The mass increase coefficient was calculated as the ratio of the pasta mass after cooking to the mass of the pasta sample weighed prior to cooking. Dry matter losses were determined using a gravimetric oven-drying method. Samples of the cooled cooking water were collected in pre-weighed evaporating dishes, which were then dried in a convection oven (WAMED, Warsaw, Poland) at 105 °C until a constant mass was achieved. The results were expressed as a percentage of loss, taking into account the mass of the pasta sample and the total volume of the cooking water remaining after pasta preparation [59].

### 3.6. Determination of the Color of Raw Materials and Pasta

The color coordinates of the raw materials and cooked pasta samples were measured using a CR-200 spectrophotometer (Konica Minolta, Tokyo, Japan) in the CIE Lab* color space. The following color parameters were determined: lightness (L*), a* (positive values indicating redness and negative values indicating greenness), and b* (positive values indicating yellowness and negative values indicating blueness). The total color difference (ΔE) between the control sample made from SE without GCF addition and the GCF-enriched pasta samples was also calculated [60].

### 3.7. Determination of Pasta Texture

The texture of pasta samples was evaluated using a TA-XT2i texture analyzer (Stable Micro Systems, London, UK) applying the Texture Profile Analysis (TPA). Immediately after cooking, the pasta samples were placed in plastic containers with a diameter of 55 mm and a height of approximately 25 mm, and subjected to two consecutive compression cycles using a cylindrical probe with a diameter of 25 mm at a test speed of 1 mm/s. The interval between the two compressions was 10 s. Texture parameters including cohesiveness, springiness, and resilience were calculated using the Texture Expert Exceed software—version 2.64 (Stable Micro Systems Ltd., Godalming, UK).

### 3.8. Determination of Antioxidant Activity and Total Phenolics

#### 3.8.1. Extract Preparation

The levels of total polyphenolic compounds and antioxidant activity were assessed in the raw materials (semolina, GCF) and in the cooked pasta samples. For extraction, 1 g of each sample was weighed and subjected to three consecutive extractions with 5 mL of 50% aqueous methanol in a laboratory shaker for 30 min, followed by centrifugation. The resulting extracts were pooled for further analyses, centrifuged again, and the supernatants were collected. The combined supernatants were stored in the dark at −20 °C.

#### 3.8.2. Determination of ABTS and DPPH Radicals Scavenging Activity

The antiradical activity was assessed based on the ability to scavenge ABTS^+^ radicals. The radical cation was generated by combining 7 mM ABTS with 2.45 mM potassium persulfate and allowing the mixture to react in the dark at room temperature for 20 h until full formation of the radical. The resulting solution was diluted to achieve an absorbance of approximately 0.70–0.72 at 734 nm. Then, 1.8 mL of the ABTS solution was mixed with 0.04 mL of the test sample, and the absorbance at 734 nm was recorded after 10 min of incubation. The percentage inhibition (PI) of ABTS discoloration was subsequently calculated as follows [61]:

(1) $$PI={\left(1-\frac{{AB}_{s}}{{AB}_{c}}\right)*100\%}_{,}$$

where AB_s_ ia absorbance of the sample and AB_c_ is absorbance of the ABTS solution.

DPPH radical scavenging activity was determined. 250 μL of DPPH solution was mixed with 10 μL of the extract, and the absorbance at 517 nm was measured after 15 min of incubation at room temperature. The percentage inhibition of DPPH discoloration was calculated according to Equation (1).

The radical-scavenging activities of the extracts against ABTS and DPPH radicals were quantified as EC_50_ values, indicating the concentration of extract (mg d.m./mL) necessary to neutralize 50% of the radicals in a dose-dependent manner [61].

#### 3.8.3. Total Polyphenols Content

The total polyphenol content was determined using the Folin–Ciocalteu method. For each sample, 0.5 mL of the methanolic extract was mixed with 0.5 mL of distilled water, 2 mL of Folin’s reagent (diluted 1:5 with water), and 2 mL of 10% Na_2_CO_3_ solution. The mixture was thoroughly mixed and allowed to react for 60 min. The absorbance was measured at 720 nm against a with 50% methanol (*v*/*v*). Total phenolic content was expressed as mg of gallic acid equivalents (mg GAE) per gram of dry mass (dm), calculated from a gallic acid standard calibration curve constructed from 10 concentration points ranging from 0 to 1 mg·mL^−1^ (R^2^ = 0.998) [61].

### 3.9. Phenolic Acids Analysis

The phenolic compounds in raw materials and cooked pasta were determined using high-performance liquid chromatography coupled with electrospray ionization tandem mass spectrometry (LC-ESI-MS/MS), following a slightly modified procedure based on the methods reported by Nowacka et al. [62] and Pietrzak et al. [63].

Separation of phenolic compounds was carried out at 25 °C, on a Zorbax SB-C18 column (2.1 × 100 mm, 1.8 mm particle size; Agilent Technologies, Santa Clara, CA, USA). The mobile phase consisted of 0.1% aqueous formic acid (solvent A) and acetonitrile with 0.1% formic acid (solvent B). The injection volume was 3 µL, and the flow rate was 300 µL/min. The gradient was changed as follows: 0–2 min—20% B; 3–4.5 min—25% B; 5.5–7 min—35%; 8–12 min—65% B; 14–16 min—80% B. The total run time was 28 min B.

The ESI-MS was operated in negative ion mode, and the parameters were as follows: capillary temperature 550 °C, curtain gas at 30 psi, nebulizer gas at 50 psi, source voltage −4500 V.

Triplicate injections were made for each standard solution and sample. The limits of detection (LOD) and quantification (LOQ) for all analytes were determined at a signal-to-noise ratio of 3:1 and 10:1, respectively. Qualitative identifications of compounds were based on comparisons of MS/MS spectra and LC retention times with the corresponding standards tested under the same conditions. The calibration curves obtained in MRM mode were used for the quantification of all analytes. Detailed conditions of LC-MS analysis are given in Table 11 and Table 12.

### 3.10. Sensory Evaluation of Pasta

Sensory evaluation was performed using a nine-point hedonic scale, with 1 representing “extremely undesirable,” 5 indicating “neither desirable nor undesirable,” and 9 denoting “extremely desirable” [64]. The study was reviewed by the Bioethics Committee (Warsaw, Poland), which determined that formal approval was not required, as the research involved only healthy adult volunteers, posed no risk of pain or discomfort, and consisted of standard sensory evaluation procedures. All participants provided informed consent, were fully informed about the product and study procedures, and could withdraw at any time. The study was conducted in accordance with the principles of the Declaration of Helsinki, ensuring participant safety and confidentiality. The panel comprised 68 untrained participants (38 women and 30 men; staff and students from the Warsaw University of Life Sciences) aged between 21 and 60 years. The panel size was chosen to provide sufficient statistical power for detecting differences in sensory attributes. Participants were selected based on self-reported good health, absence of gluten allergies, and regular pasta consumption. The assessed characteristics included taste and aroma, color, texture, and overall acceptability. Evaluations were conducted under white lighting in a room maintained at 22 °C. Samples were presented in a randomized order and coded to ensure blinding so that participants were unaware of the sample identity.

### 3.11. Statistical Analysis

Statistical analyses were performed using Statistica 13.3 (TIBCO Software, Palo Alto, CA, USA). Two-way ANOVA was applied for the main experiments, and one-way ANOVA was used to compare the chemical and physical properties of semolina (SE) and green coffee flour (GCF), with homogeneous groups identified by Tukey’s test (α = 0.05). All determinations were carried out in triplicate. Principal component analysis (PCA) was also conducted to examine relationships among the variables.

## 4. Conclusions

The study demonstrated that both the addition of GCF and the drying method significantly affected the evaluated properties of pasta. While GCF had no impact on the microstructure of convectively dried pasta, microwave-vacuum drying induced visible cracks and a heterogeneous starch–protein matrix even at a 2% addition level. Microwave-vacuum-dried samples exhibited a substantially shorter optimal cooking time and higher water absorption compared to convectively dried pasta, whereas increasing the GCF content prolonged cooking time and elevated cooking losses. Furthermore, convectively dried samples appeared darker than microwave-vacuum-dried ones, with higher GCF levels leading to increased darkening and redness. Convectively dried pasta showed reduced elasticity and cohesiveness with increasing GCF addition, whereas microwave-vacuum-dried pasta maintained a consistent texture regardless of GCF content. The inclusion of GCF enhanced the antioxidant capacity of pasta, with the most pronounced effect observed at 2% supplementation, while higher levels produced diminished benefits; the drying method had minimal influence on phenolic content and antioxidant activity. Fortification with GCF also increased the content of phenolic acids, particularly chlorogenic acid and its isomers, with convectively dried pasta generally exhibiting higher levels than microwave-vacuum-dried samples. The highest overall consumer acceptability was observed for convectively dried pasta without GCF and for samples supplemented with 2%, whereas pasta with higher GCF levels (8% in convective drying and 6–8% in microwave-vacuum drying) was poorly accepted. In summary, convectively dried pasta fortified with 2% GCF demonstrated superior quality and potential health-promoting properties compared to pasta obtained via microwave-vacuum drying. Therefore, GCF-fortified pasta has potential applications in the development of functional foods aimed at promoting health through natural bioactive compounds. Future studies could explore the combination of GCF with other plant-based additives, such as fruit or vegetable powders, to further enhance nutritional value, diversify sensory profiles, and optimize consumer acceptability.

## Figures and Tables

**Table 1:** Basic chemical composition of raw materials.

Sample	Protein (% d.m.)	Ash (% d.m.)	Fat (% d.m.)	Fiber (% d.m.)	Carbohydrates (% d.m.)
SE	12.01 ± 0.05 ^A^	0.87 ± 0.01 ^A^	0.80 ± 0.03 ^A^	3.25 ± 0.06 ^A^	83.07 ± 0.03 ^A^
GCF	17.59 ± 0.00 ^B^	3.37 ± 0.04 ^B^	10.45 ± 0.12 ^B^	22.05 ± 0.18 ^B^	46.54 ± 0.03 ^B^

Different letters (^A,B^) indicate significant differences between groups; SE—semolina, GCF—green coffee flour.

**Table 2:** Culinary properties of control and enriched pasta.

Drying Method	Green Coffee Flour Content (%)	Optimal Cooking Time (min)	Weight Increase Index (-)	Cooking Loss (%)
Convective	0	7.0 ± 0.05 ^e^	2.5 ± 0.0 ^a^	5.10 ± 0.00 ^a^
	2	8.5 ± 0.00 ^f^	2.6 ± 0.1 ^ab^	5.30 ± 0.01 ^b^
	4	9.0 ± 0.10 ^g^	2.6 ± 0.1 ^ab^	5.98 ± 0.01 ^c^
	6	10.5 ± 0.05 ^h^	2.8 ± 0.0 ^c^	6.88 ± 0.02 ^d^
	8	11.5 ± 0.00 ^i^	2.8 ± 0.0 ^c^	7.31 ± 0.04 ^e^
Microwave-Vacuum	0	3.5 ± 0.00 ^a^	3.0 ± 0.0 ^d^	7.54 ± 0.02 ^f^
	2	4.0 ± 0.10 ^b^	2.9 ± 0.0 ^cd^	7.75 ± 0.00 ^g^
	4	4.5 ± 0.10 ^c^	3.0 ± 0.1 ^d^	8.05 ± 0.02 ^h^
	6	5.5 ± 0.05 ^d^	3.0 ± 0.1 ^d^	9.08 ± 0.00 ^i^
	8	5.5 ± 0.00 ^d^	3.3 ± 0.1 ^e^	10.40 ± 0.00 ^j^
Factor		Two-factor analysis of variance		
		*p*-value		
Drying method (DM)		<0.001	<0.001	<0.001
Green coffee flour (GCF) content		<0.001	<0.001	<0.001
DM × GCF		<0.001	<0.001	<0.001

Different letters (^a–j^) indicate significant differences between groups (*p* < 0.05).

**Table 3:** Color of raw materials.

Sample	L* (-)	a* (-)	b* (-)
SE	84.57 ± 0.35 ^B^	1.66 ± 0.18 ^A^	22.31 ± 0.41 ^B^
GCF	71.65 ± 0.47 ^A^	2.16 ± 0.12 ^B^	16.42 ± 0.24 ^A^

Different letters (^A,B^) indicate significant differences between groups; SE—semolina, GCF—green coffee flour.

**Table 4:** Color parameters of control and enriched pasta.

Drying Method	Green Coffee Flour Content (%)	L* (-)	a* (-)	b* (-)	ΔE (-)
Convective	0	83.30 ± 0.69 ^d^	0.92 ± 0.03 ^ab^	16.51 ± 0.66 ^g^	-
	2	81.18 ± 0.43 ^c^	0.99 ± 0.05 ^bc^	15.46 ± 0.36 ^ef^	2.37 ^b^
	4	78.71 ± 0.57 ^b^	1.21 ± 0.06 ^d^	15.27 ± 0.32 ^def^	4.76 ^d^
	6	77.88 ± 0.55 ^b^	1.40 ± 0.15 ^ef^	14.69 ± 0.30 ^cde^	5.74 ^e^
	8	76.23 ± 0.43 ^a^	1.56 ± 0.09 ^f^	14.48 ± 0.36 ^cd^	7.37 ^f^
Microwave-vacuum	0	83.06 ± 0.47 ^d^	0.78 ± 0.03 ^a^	15.83 ± 0.60 ^fg^	-
	2	82.37 ± 0.37 ^d^	0.96 ± 0.06 ^bc^	14.06 ± 0.44 ^bc^	1.91 ^a^
	4	80.65 ± 0.69 ^c^	1.13 ± 0.08 ^cd^	13.90 ± 0.39 ^bc^	3.12 ^c^
	6	78.44 ± 0.52 ^b^	1.29 ± 0.11 ^de^	13.49 ± 0.46 ^ab^	6.01 ^ef^
	8	78.01 ± 0.19 ^b^	1.45 ± 0.08 ^ef^	12.64 ± 0.32 ^a^	7.85 ^f^
Factor		Two-factor analysis of variance			
		*p*-value			
Drying method (DM)		<0.001	<0.001	<0.001	<0.001
Green coffee flour (GCF) content		<0.001	<0.001	<0.001	<0.001
DM × GCF		<0.001	0.598	0.077	<0.001

Different letters (^a–g^) indicate significant differences between groups (*p* < 0.05).

**Table 5:** Texture parameters of control and enriched pasta.

Drying Method	Green Coffee Flour Content (%)	Elasticity (-)	Springiness (-)	Cohesiveness (-)
Convective	0	0.35 ± 0.02 ^c^	0.84 ± 0.01 ^c^	0.71 ± 0.01 ^f^
	2	0.30 ± 0.00 ^c^	0.74 ± 0.01 ^bc^	0.69 ± 0.01 ^ef^
	4	0.23 ± 0.00 ^b^	0.51 ± 0.02 ^ab^	0.57 ± 0.02 ^c^
	6	0.20 ± 0.04 ^ab^	0.49 ± 0.03 ^b^	0.43 ± 0.03 ^b^
	8	0.15 ± 0.01 ^a^	0.39 ± 0.05 ^a^	0.36 ± 0.05 ^a^
Microwave-vacuum	0	0.23 ± 0.00 ^b^	0.62 ± 0.00 ^abc^	0.61 ± 0.00 ^cd^
	2	0.21 ± 0.01 ^ab^	0.56 ± 0.18 ^ab^	0.63 ± 0.18 ^cde^
	4	0.23 ± 0.02 ^b^	0.57 ± 0.01 ^ab^	0.66 ± 0.01 ^def^
	6	0.23 ± 0.02 ^b^	0.52 ± 0.00 ^ab^	0.64 ± 0.00 ^de^
	8	0.23 ± 0.01 ^b^	0.59 ± 0.02 ^ab^	0.61 ± 0.02 ^cd^
Factor		Two-factor analysis of variance		
		*p*-value		
Drying method (DM)		<0.001	<0.001	<0.001
Green coffee flour (GCF) content		0.025	0.485	<0.001
DM × GCF		<0.001	0.004	<0.001

Different letters (^a–f^) indicate significant differences between groups (*p* < 0.05).

**Table 6:** Total polyphenols and antioxidant capacity of raw materials.

Sample	TPC (mg GAE/g d.m.)	EC_50ABTS_ (mg d.m./mL)	EC_50DPPH_ (mg d.m./mL)
SE	0.729 ± 0.08 ^A^	127.17 ± 1.63 ^A^	315.23 ± 3.62 ^A^
GCF	44.90 ± 0.65 ^B^	8.77 ± 0.06 ^B^	10.47 ± 0.25 ^B^

Different letters (^A,B^) indicate significant differences between groups; SE—semolina, GCF—green coffee flour, TPC—total polyphenols content; EC_50_—effective concentration of the extract required to reduce 50% of ABTS and DPPH free radicals, respectively.

**Table 7:** Total polyphenols and antioxidant capacity of control and enriched pasta.

Drying Method	Green Coffee Flour Content (%)	TPC (mg GAE/g d.m.)	EC_50ABTS_ (mg d.m./mL)	EC_50DPPH_ (mg d.m./mL)
Convective	0	0.49 ± 0.02 ^a^	138.57 ± 0.35 ^g^	322.27 ± 1.80 ^e^
	2	1.14 ± 0.01 ^b^	46.03 ± 1.33 ^e^	149.47 ± 0.75 ^d^
	4	1.91 ± 0.08 ^c^	40.07 ± 0.74 ^cd^	120.43 ± 1.17 ^c^
	6	2.36 ± 0.01 ^d^	36.93 ± 0.51 ^b^	96.83 ± 0.32 ^b^
	8	3.09 ± 0.06 ^e^	31.57 ± 0.81 ^a^	81.20 ± 0.70 ^a^
Microwave-vacuum	0	0.46 ± 0.01 ^a^	142.80 ± 1.51 ^h^	329.67 ± 3.07 ^f^
	2	1.02 ± 0.02 ^b^	49.17 ± 1.23 ^f^	152.97 ± 3.35 ^d^
	4	1.89 ± 0.02 ^c^	41.80 ± 0.70 ^d^	116.47 ± 0.93 ^c^
	6	2.20 ± 0.06 ^d^	37.83 ± 0.78 ^bc^	93.73 ± 1.79 ^b^
	8	2.93 ± 0.04 ^e^	32.50 ± 0.70 ^a^	77.67 ± 0.74 ^a^
Factor		Two-factor analysis of variance		
		*p*-value		
Drying method (DM)		<0.001	<0.001	0.087
Green coffee flour (GCF) content		<0.001	<0.001	<0.001
DM × GCF		0.018	0.022	0.022

Different letters (^a–h^) indicate significant differences between groups (*p* < 0.05); TPC—total polyphenols content, EC_50_—effective concentration of the extract required to reduce 50% of ABTS and DPPH free radicals, respectively.

**Table 8:** Phenolic acids content (ng/g d.m.) in raw material samples.

Sample	Gallic	Protocate Chuic	*p*-Coumaric	Salicylic	Chlorogenic	Crypto -Chlorogenic	Neochlorogenic	Caffeic
SE	nd	Trace	200 ± 1 ^A^	trace	58,467 ± 701 ^A^	2871 ± 19 ^A^	2618 ± 10 ^A^	trace
GCF	317 ± 6	1997 ± 9	2973 ± 28 ^B^	648 ± 6	224,437 ± 4734 ^B^	91,460 ± 808 ^B^	165,477 ± 2831 ^B^	191,500 ± 527

Different letters (^A,B^) indicate significant differences between groups; SE—semolina, GCF—green coffee flour, nd—no detection.

**Table 9:** Phenolic acids content (ng/g d.m. in control of control and enriched pasta.

DM	GCF (%)	Gallic	Protocate- Chuic	*p*-Coumaric	Chlorogenic	Neochloro- Genic	Cryptochlorogenic	Caffeic
Convective	0	trace	nd	327 ± 4 ^a^	58,063 ± 1045 ^a^	2503 ± 222 ^a^	2867 ± 96 ^a^	trace
	2	42 ± 1 ^c^	886 ± 8 ^a^	692 ± 13 ^c^	184,319 ± 1799 ^c^	35,533 ± 311 ^c^	23506 ± 83 ^c^	12,792 ± 244 ^c^
	4	53 ± 2 ^d^	1007 ± 3 ^c^	773 ± 9 ^d^	213,004 ± 1994 ^f^	65,723 ± 891 ^d^	33,020 ± 285 ^d^	13,269 ± 48 ^e^
	6	86 ± 1 ^e^	1061 ± 7 ^d^	843 ± 8 ^e^	212,956 ± 1268 ^f^	65,861 ± 191 ^f^	37,216 ± 353 ^f^	13,962 ± 104 ^f^
	8	93 ± 2 ^f^	1077 ± 16 ^d^	903± 4 ^f^	211,366 ± 2174 ^f^	72,670 ± 306 ^g^	42,166 ± 312 ^h^	14,610 ± 189 ^d^
Microwave -vacuum	0	trace	nd	303 ± 4 ^a^	60,352 ± 151 ^a^	2508 ± 50 ^a^	2717 ± 49 ^a^	trace
	2	34 ± 1 ^b^	trace	567 ± 8 ^b^	177,167 ± 1041 ^b^	26,816 ± 509 ^b^	20,762 ± 411 ^b^	7915 ± 28 ^a^
	4	45 ± 2 ^c^	892 ± 7 ^a^	625 ± 18 ^c^	196,759 ± 1641 ^d^	54,130 ± 476 ^d^	30,419 ± 495 ^d^	8244 ± 144 ^a^
	6	30 ± 0 ^a^	956 ± 10 ^b^	790 ± 5 ^d^	199,705 ± 388 ^de^	50,946 ± 259 ^e^	32,378 ± 184 ^e^	10,632 ± 115 ^b^
	8	29 ± 1 ^a^	971 ± 6 ^b^	798 ± 12 ^d^	203,421 ± 2758 ^e^	79,355 ± 655 ^h^	39,911 ± 192 ^g^	10,567 ± 182 ^b^
Factor		Two-factor analysis of variance *p-*value						
DM		<0.001	<0.001	<0.001	<0.001	<0.001	<0.001	<0.001
GCF		<0.001	<0.001	<0.001	<0.001	<0.001	<0.001	<0.001
DM × GCF		<0.001	<0.001	<0.001	<0.001	<0.001	<0.001	<0.001

Different letters (^a–h^) indicate significant differences between groups (*p* < 0.05); DM—drying method, GCF—green coffee flour, nd—no detection.

**Table 10:** Results of pasta sensory evaluation.

Drying Method	GCF (%)	Taste	Smell	Color	Texture	OA
Convective	0	8.3 ± 1.1 ^d^	8.2 ± 0.7 ^c^	8.5 ± 0.5 ^e^	8.3 ± 1.5 ^d^	8.3 ± 0.9 ^d^
	2	6.7 ± 1.5 ^bcd^	6.9 ± 1.9 ^bc^	6.1 ± 1.6 ^cd^	7.3 ± 1.3 ^cd^	6.8 ± 0.9 ^cd^
	4	5.5 ± 1.7 ^abc^	6.3 ± 2.0 ^abc^	5.4 ± 1.8 ^bcd^	6.1 ± 1.3 ^bcd^	5.8 ± 1.2 ^bc^
	6	4.8 ± 2.3 ^abc^	5.5 ± 1.9 ^ab^	4.5 ± 2.1 ^abc^	5.1 ± 1.8 ^abc^	5.0 ± 1.6 ^ab^
	8	3.5 ± 2.5 ^a^	4.4 ± 2.5 ^a^	3.2 ± 1.9 ^a^	4.7 ± 1.9 ^ab^	3.9 ± 1.7 ^a^
Microwave-vacuum	0	7.0 ± 2.0 ^cd^	6.1 ± 1.8 ^abc^	6.7 ± 2.4 ^de^	4.5 ± 2.8 ^ab^	6.1 ± 1.7 ^bc^
	2	5.6 ± 2.0 ^abc^	6.5 ± 1.9 ^abc^	4.7 ± 1.9 ^abcd^	4.5 ± 2.5 ^ab^	5.3 ± 1.5 ^abc^
	4	4.7 ± 1.6 ^ab^	6.1 ± 1.8 ^abc^	4.3 ± 1.9 ^abc^	4.7 ± 2.4 ^ab^	5.0 ± 1.5 ^ab^
	6	3.3 ± 1.8 ^a^	4.7 ± 2.2 ^ab^	3.8 ± 1.8 ^ab^	3.4 ± 2.4 ^a^	3.8 ± 1.5 ^a^
	8	3.6 ± 2.8 ^a^	4.9 ± 2.6 ^ab^	3.5 ± 1.8 ^ab^	3.5 ± 2.3 ^a^	3.9 ± 1.8 ^a^
Factor		Two-factor analysis of variance *p-*value				
Drying method (DM)		<0.001	<0.001	<0.001	<0.001	<0.001
Green coffee flour (GCF) content		0.006	0.062	0.002	<0.001	<0.001
DM × GCF		0.623	0.167	0.213	0.091	0.075

Different letters (^a–e^) indicate significant differences between groups (*p* < 0.05); GCF—green coffee flour, OA—overall acceptability.

**Table 11:** LC-ESI-MS/MS analytical results of phenolic acids investigated in samples. Compounds confirmed by comparison with authentic standards.

Compound	Retention Time (min)	[M-H]^−^ (*m*/*z*)	Fragment Ions (*m*/*z*)	Colision Energy (eV)
Phenolic acids				
Gallic acid	5.16	168.7	78.9 124.9	−36 −14
3-*O*-caffeoylquinic acid (neochlorogenic acid)	6.9	353	191 178.9	−3 −3
Protocatechuic acid	8.42	152.9	80.9 107.8	−26 −38
5-caffeoylquinic acid (chlorogenic acid)	9.30 10.42	352.9	190.8 84.9	−24 −60
4-caffeoylquinic acid (cryptochlorogenic acid)	9.4	353	173 135	−3 −3
4-Hydroxybenzoic acid	10.84	136.8	92.9	−18
Caffeic acid	11.38	178.7	88.9 134.9	−46 −16
Syringic acid	11.42	196.9	122.8 181.9	−24 −12
4-Hydroxycinnamic acid (*p*-coumaric acid)	14.10	162.7	119 93	−14 −44
Ferulic acid	14.80 15.22	192.8	133.9 177.9	−16 −12
Salicylic acid	17.91	136.8	93 75	−16 −48

**Table 12:** Limit of detection (LOD), limit of quantification (LOQ), and calibration curve parameters for phenolic acids.

Compound	LOD (ng/mL)	LOQ (ng/mL)	R^2^	Linearity Range (ng/ mL)
Phenolic acids				
Gallic acid	10	20	0.9995	20–18,500
3-*O*-caffeoylquinic acid (neochlorogenic acid)	20	40	0.9996	40–10,000
Protocatechuic acid	200	400	0.9988	1890–18,900
5-caffeoylquinic acid (chlorogenic acid)	72	180	0.9991	180–18,000
4-caffeoylquinic acid (cryptochlorogenic acid)	20	40	0.9979	40–4000
4-Hydroxybenzoic acid	200	250	0.9994	700–19,250
Caffeic acid	195	389	0.9991	389–19,500
Syringic acid	500	732	0.9993	732–18,300
4-Hydroxycinnamic acid (*p*-coumaric acid)	83	200	0.9990	400–13,800
Ferulic acid	1250	1830	0.9985	1830–36,500
Salicylic acid	500	600	0.9984	600–18,000

## Data Availability

The data presented in this study are available on request from the corresponding author.
